# Association of urinary activity of MMP-9 with renal impairment in Mexican patients with type 2 diabetes mellitus

**DOI:** 10.7717/peerj.6067

**Published:** 2018-12-06

**Authors:** Alan Uriel García-Tejeda, Clara Luz Sampieri, Irene Suárez-Torres, Jaime Morales-Romero, Verónica Patricia Demeneghi-Marini, Magda Elena Hernández-Hernández, Arturo Rodríguez-Hernández

**Affiliations:** 1 Instituto de Salud Pública, Universidad Veracruzana, Xalapa, Veracruz, México; 2 Instituto Mexicano del Seguro Social, Unidad de Medicina Familiar No. 66. Delegación Veracruz Norte, Xalapa, Veracruz, México; 3 Secretaría de Salud del Estado de Veracruz, Unidad de Inteligencia Epidemiológica en Salud, Xalapa, Veracruz, México; 4 Instituto Mexicano del Seguro Social, Unidad de Medicina Familiar No. 10, Xalapa, Veracruz, México

**Keywords:** Renal impairment, Urine MMP-9, Matrix metalloproteinases, Type 2 diabetes mellitus

## Abstract

**Background:**

Diabetic kidney disease is the most common cause of chronic kidney disease (CKD). An early event in diabetic kidney disease is alteration of the glomerular basement membrane and the mesangial expansion. Matrix metalloproteinases (MMP) are a family of endopeptidases responsible for controlling the pathophysiological remodeling of tissues, including renal tissues. MMP-9 in human urine has been proposed as a marker of diabetic nephropathy and urinary tract infections (UTI).

**Methods:**

A cross-sectional study was conducted in type 2 diabetes mellitus (T2DM) patients who receive first level medical attention in Mexico. We used ELISA to measure MMP-9 levels in the urine of subjects with T2DM ≥ 18 years of age, who fulfilled the clinical requirements for calculation of glomerular filtration rate (GFR), according to the K/DOQI guide, in an attempt to identify whether MMP-9 levels in T2DM differ in patients with and without renal impairment. Univariate and multivariable analyses were performed in order to identify the association between MMP-9 and renal impairment.

**Results:**

Included in the study were 34 (45%) subjects with renal impairment and 42 (55%) without. In the group with renal impairment, 10 subjects corresponded to stages 1–2 and 24 subjects corresponded to stage 3, according to their values of GFR and urinary albumin, following that proposed by the K/DOQI. No differences were found relating to sex, age, having or not having a partner, education, being able to read and write a message and duration of T2DM. Moreover, no differences were found between the groups in terms of weight, height, body mass index, waist size in general and frequency of UTI. In contrast, serum creatinine and urinary albumin were higher in the group with renal impairment, while GFR was greater in the group without renal impairment. Levels of MMP-9 were greater in women compared to men. Through univariate analysis in the general population, the presence of MMP-9 and that of its percentile 90 (P_90_) P_90_ were associated with the renal impairment group; however, in patients without UTI, only the presence of MMP-9 was associated with the renal impairment group, and no association was found with its P_90_. Multivariate analysis revealed an association between MMP-9 and its P_90_ with renal impairment.

**Discussion:**

It is necessary to validate sensitive and non-invasive biological markers of CKD. We demonstrate that the presence and P_90_ of urinary MMP-9 are associated with renal impairment in Mexican patients with T2DM. While high levels of MMP-9 were associated to females and UTI, the presence of UTI was not associated with the incidence of renal impairment.

## Introduction

Diabetic kidney disease is now the most common cause of chronic kidney disease (CKD), which has become a major public health problem worldwide (Ghaderian et al., 2015; Gheith et al., 2016). An early event in diabetic kidney disease is alteration of the glomerular basement membrane and the mesangial expansion, which may result in microalbuminuria, subsequent proteinuria and eventual end stage renal disease (Morais et al., 2005; Thrailkill, Clay Bunn & Fowlkes, 2009). Renal interstitial fibrosis is considered to be the final outcome of all CKD, regardless of etiology (Pei et al., 2014). Chronic progressive renal fibrosis remains an unresolved challenge in the management of diabetic nephropathy, since it is still difficult to identify at early stages due to the lack of noninvasive predictive biomarkers (Zheng et al., 2012). A major limitation to the use of albuminuria as a marker in diabetic kidney disease is its sensitivity. It has been reported that low glomerular filtration rate (GFR) is present in half or more without increased albuminuria (Tuttle et al., 2014). Moreover, in half or more patients urinary loss underestimates the potential of the kidney to clear albumin since it is hydrolyzed with the amino acid and returned to the plasma (Levitt & Levitt, 2016). In addition, the characteristics of equations used to estimate GFR make them significantly less precise at higher GFR. This is of particular concern in diabetic kidney disease, which may be associated with hyperfiltration (Tuttle et al., 2014). It has been proposed that an ideally non-invasive biomarker, involved in the early diagnosis of diabetic kidney disease and related to the rate of extracellular matrix remodeling process, would provide the best marker of progression (Altemtam, Nahas & Johnson, 2012).

Matrix metalloproteinases (MMP) are a large family of zinc and calcium-dependent endopeptidases that collectively degrade all components of the extracellular matrix and basement membrane proteins, and are thus responsible for controlling the pathophysiological remodeling of tissues (Pulido-Olmo et al., 2016). In addition to their role in extracellular matrix turnover, MMPs release or activate several growth factors, including IGF-II, HBEGF, TNF-α and TGF-β, that are implicated in renal hypertrophy, tubular cell proliferation and renal fibrosis (Thrailkill, Clay Bunn & Fowlkes, 2009). In recent years, new paradigms have emerged in which the extracellular matrix turnover governed by MMPs is a hallmark of diverse pathological and generalized states such as oxidative stress, apoptosis, endothelial-mesenchymal transition and inflammation (Schulz, 2007; Hua & Nair, 2015; Zhao et al., 2017). Moreover, in animal models, Mmp-11 (Arcidiacono et al., 2017), MMP-2 (Miksztowicz et al., 2014) and MMP-9 (Miksztowicz et al., 2014) have been implicated in insulin resistance states.

The *MMP-9* (gelatinase-B, macrophage gelatinase or neutrophil gelatinase) mRNA in human urinary sediment has been proposed as a marker of epithelial-mesenchymal transition, which correlates with urinary albumin excretion and blood urea nitrogen and presents a high diagnostic value for diabetic nephropathy, defined by authors as at least 5 years from diagnosis of type 2 diabetes mellitus (T2DM), presence of diabetic retinopathy, elevated albumin/creatinine ratio and absence of other kidney disease (Zheng et al., 2012). It has been proposed that urinary MMP-9 may be useful for evaluating the degree of renal injuries in patients with diabetic nephropathy, especially in the early stage (Tashiro et al., 2004). Moreover, in Zucker diabetic rats, *MMP-9* expression is up-regulated in renal parietal epithelial cells and its expression is associated with loss of adjacent podocytes (Zhang et al., 2015). Albumin in renal parietal epithelial cells induces *MMP-9* expression and secretion (Zhang et al., 2015). The potentially protective role of functional polymorphisms of *MMP-9* has been associated with the prevalence of CKD in the Japanese population (Okada et al., 2012); however, to our knowledge, there have been no similar studies conducted in the Mexican population.

Bacterial infections of the urinary tract infections (UTI) are considered to be the most common bacterial infection in general (Foxman, 2003). Defense against UTI is dependent upon on neutrophils (Anderson et al., 2003). MMP-9 secretion by neutrophils allows these cells to cross the epithelial basement membrane in order to combat UTI infection (Chin & Parkos, 2006; Schiwon et al., 2014). In UTI of *MMP-9*^−/−^ mice infected with uropathogenic *Escherichia coli*, neutrophils were found to accumulate underneath the uroepithelium and did not enter it (Zec et al., 2016). Here, we measured MMP-9 levels in urine samples of subjects with T2DM in an attempt to identify whether these levels differ in patients with and without renal impairment.

## Methods

### Design and study population

A cross-sectional study was conducted (March–May 2015) in T2DM patients that receive first level medical attention at the *Unidad de Medicina Familiar Número 66* at the *Instituto Mexicano del Seguro Social*. Criteria for inclusion in the study were: ≥18 years and fulfillment of the clinical requirements for calculation of GFR, according to the guide K/DOQI (National Kidney Foundation, 2002). Subjects undergoing menstruation, hematuria, antibiotic treatment and those who had had intercourse in the 72 h preceding the clinical sampling were excluded.

### Calculation of sample and sampling

The medical unit in which this study was conducted has 28 consultancies for family medical issues in morning shifts from 8.00 to 14.00 h, and afternoon shifts from 14.00 to 20.00 h. In order to randomly select subjects, sampling was conducted in three stages: in the first, each shift was considered a stratum; in the second, two consultancies were selected daily at random in each stratum; and the third stage included all patients with T2DM, who consecutively attended a medical consultation in the consultancies selected in the previous stage, and who fulfilled the criteria of selection.

### Ethics

The research protocol was approved by *Instituto Mexicano del Seguro Social* (Register No. R-2015-3004-4). This study was conducted according to the principles of the Declaration of Helsinki. The subjects who agreed to participate all signed an informed consent.

### Clinical, biochemical and microbiological assessments

Blood samples were taken, following 8 h of fasting, and anthropometric and vital sign measurements recorded. Tubes without anticoagulant (Vacutainer 368175) were used for serum collection. Creatinine content in the serum was determined (ERBA Mannheim Clinical Chemistry Analizer XL200; BioSystems Laboratories, Mumbai, India). Morning midstream urine specimens were collected for analysis with Multistix 10 SG® urine reagent strip, with albumin determination (ERBA Mannheim Clinical Chemistry Analizer XL200; BioSystems Laboratories, Mumbai, India) and microbiological evaluation by aerobic bacterial cultures, using cystine-lactose-electrolyte deficient (CLED), MacConkey and Mannitol salt agar.

### MMP-9 quantification

Aliquots of urine samples were centrifuged at 2,000*g* for 10 min at 4 °C (Altemtam, Nahas & Johnson, 2012), stored at −80 °C for less than 60 days and defrosted on a single occasion only. Total urine MMP-9 concentration was measured using a commercially available enzyme-linked immunosorbent assay kit (Quantikine; R&D systems, Minneapolis, MN, USA). Each sample was assayed in duplicate. Blind analysis of the samples was conducted. According to the provider, the ELISA kit employed has a sensitivity of 0.156 ng/mL and recognizes both pro and active forms. Intra and inter-assay coefficients of variation were found to be 5.9% and 7.9%, respectively.

### Variables

The GFR was estimated according to the K/DOQI guide, (National Kidney Foundation, 2002) with classification as “no renal impairment” with GFR ≥60 mL/min/1.73 m^2^ and ≤2.9 mg/dL urinary albumin, or “with renal impairment” with GFR ≤59 mL/min/1.73 m^2^ and ≥3.0 mg/dL urinary albumin or with GFR ≤59 mL/min/1.73 m^2^, regardless of the level of urinary albumin. T2DM status was assigned with use of insulin or hypoglycemic agents or ≥126 mg/dL serum glucose under fasting or report of medical diagnosis. Central obesity (CO) with ≥81 cm waist size for females and ≥91 cm for males. Overweightness (OW) with body mass index (BMI) ≥25 kg/m^2^. Normoalbuminuria with ≤2.9 mg/dL urinary albumin. Microalbuminuria with 3–29 mg/dL urinary albumin. Macroalbuminuria with ≥30 mg/dL urinary albumin. UTI for women with self report of burning sensation on urination and ≥10^2^ colony-forming unit (CFU) per milliliter of a single uropathogen (Bent et al., 2002; Scottish Intercollegiate Guidelines Network (SIGN), 2012) or ≥10^5^ CFU per milliliter of a single uropathogen (Bent et al., 2002; Scottish Intercollegiate Guidelines Network (SIGN), 2012). UTI for men with self report of burning sensation on urination and ≥10^3^ CFU per milliliter of a single uropathogen (Scottish Intercollegiate Guidelines Network (SIGN), 2012) or ≥10^5^ CFU per milliliter of a single uropathogen (Scottish Intercollegiate Guidelines Network (SIGN), 2012). Vigorous exercise was defined as the practice of athletics, tennis, swimming or soccer. Occupation was classified as: housewife, other occupation (employee, retired, merchant, taxi driver, farmer, childcarer and priest) and no occupation.

### Statistical analysis

Categorical variables were summarized using absolute frequencies and percentages; continuous variables of non-normal distribution were expressed through medians and the interquartile range. Proportions were compared by chi-square test or Fisher’s exact test, and medians were compared by Mann–Whitney *U*-test. MMP-9 was classified as *present* (>0 ng/mL) or *absent* (zero ng/mL) according to the 90th percentile (P_90_). It should be noted that, due to the sensitivity of the ELISA kit, there is a possibility for the production of false negatives in this study. The correlation analysis was conducted with the Spearman’s rank correlation. Multivariate analysis was performed in order to identify the association between MMP-9 (independent variable) and renal impairment (dependent variable). Four models were constructed in which the possible covariates were: age >60 years, presence of UTI, presence of nitrites in urine determinate by reagent strip, duration of T2DM (≥15 years) and sex (male). The ORs and CI95% were calculated by logistic regression. A value of *P* ≤ 0.05 was considered statistically significant. EpiDat version 3.1 and IBM-SPSS Statistics, version 22.0 (Armonk, NY, USA) were used for the analyses.

## Results

A total of 120 patients were invited to participate in the study, with signed informed consent obtained from 90% of the invitees. A total of 109 subjects were then weighed, measured and their urine samples analyzed, with 33 subjects excluded for hematuria. The total number of subjects finally included in the study was 76.

### Characteristics of the population

Of the 76 subjects included, 34 corresponded to the group with renal impairment (45%) and 42 (55%) to that of no renal impairment. In the group with renal impairment, one subject corresponded, according to their values of GFR and urinary albumin, to that proposed by the K/DOQI as guide stage 1; with nine subjects at stage 2 and 24 subjects at stage 3.

Only one subject in the no renal impairment group reported the current consumption of tobacco, the rest of the population (*n* = 75) reported that they did not smoke. In total, 65 subjects reported no practice of vigorous exercise, 33/42 (79%) of whom corresponded to the group with no renal impairment and 32/34 (94%) to that renal impairment; the rest of the subjects reported exercising vigorously very few times or sometimes. There were no differences between the groups.

On comparison of sex, age, having or not having a partner, education, being able to read and write a message and duration of T2DM, systemic arterial hypertension there were no differences between the groups (Table 1). Within the population in general, a considerable percentage were housewives (50%) and only 4% declared not having an occupation (Table 1). Metformin, glibenclamide, insulin, acarbose and pliogitazone were prescribed in 86%, 45%, 21%, 12% and 9%, respectively in the group with renal impairment and in 79%, 56%, 18%, 9% and 18%, respectively in the group without renal impairment, there were no differences between the groups. Most patients were receiving treatment for T2DM involving at least two medications (Table 1**)**.

**Table 1:** General characteristics of 76 participants according to renal impairment status.

Characteristic	General *n* = 76		No renal impairment *n* = 42		With renal impairment *n* = 34		*P*-value
	*n*	%	*n*	%	*n*	%	
**Sex**							
Female	52	(68)	26	(62)	26	(76)	0.17
Male	24	(32)	16	(38)	8	(24)	
Age (years)	60.67	[9.59]	59.38	[10.37]	62.26	[8.41]	0.194
T2DM (years)	12.58	[7.78]	11.56	[8.07]	13.84	[7.34]	0.21
**T2DM therapy**							
No medication	6	(8)	4	(10)	2	(6)	NA
Monotherapy	22	(29)	12	(29)	10	(29)	
Double therapy	40	(53)	20	(48)	20	(59)	
Triple therapy	8	(11)	6	(14)	2	(6)	
**SAT**							
Yes	48	(63)	26	(62)	22	(65)	0.80
No	28	(37)	16	(38)	12	(35)	
**SAT therapy**							
Monotherapy	24	(50)	15	(58)	9	(41)	NA
Double therapy	21	(44)	11	(42)	10	(45)	
Triple therapy	3	(6)	0	(0)	3	(14)	
**Marital status**							
With partner	44	(58)	27	(64)	17	(50)	0.21
Without partner	32	(42)	15	(36)	17	(50)	
**Occupation**							
Housewife	38	(50)	19	(45)	19	(56)	NA
Other	35	(46)	20	(48)	15	(44)	
No occupation	3	(4)	3	(7)	0	(0)	
**Education**							
Lower than primary	14	(18)	8	(19)	6	(18)	0.88
Primary or above	62	(82)	34	(81)	28	(82)	
**Able to read and write a message**							
No	4	(5)	1	(2)	3	(9)	0.32
Yes	72	(95)	41	(98)	31	(91)	
Weight (kg)*	68.12	[12.31]	69.04	[12.34]	66.86	[12.37]	0.47
Height (cm)*	152	146, 158	155	146, 163	152	148, 155	0.37
BMI (kg/m^2^)*	28.71	[3.91]	28.71	[3.73]	28.71	[4.21]	0.99
WS general^†^	92.25	88.0, 102.0	92.0	88.0, 102.0	95.0	85.5, 102.5	0.77
WS female^†^	91.0	86.0, 100.6	91.0	87.0, 96.7	92.0	81.0, 103.0	0.93
WS male^†^	97.5	89.0, 106.0	99.5	88.0, 107.0	107.0	96.5, 91.2	0.97
SC general^‡^	0.96	0.25	0.85	0.20	1.10	0.25	<0.001
SC female^‡^	0.89	0.24	0.75	0.15	1.04	023	<0.001
SC male^‡^	1.10	0.23	1.0	0.17	1.31	0.21	<0.001
UAlb^‡^	1.40	0.70, 3.15	1.00	0.60, 1.80	3.60	1.15, 5.85	<0.001
GFR^§^	69.67	56.48, 87.05	83.89	69.13, 95.23	56.00	49.90, 60.55	<0.001
CFU^╗^	550	0, 5,000	6,000	0, 5,850	500	100, 5,000	0.80
Glu^‡^	160	124,188	159	124, 183	163	132, 193	0.708

**Notes:**

Values for the categorical variables are presented as frequency (percentage) or median and interquartile range percentile 25, percentile 75; age and T2DM duration as mean [±standard deviation]. All percentages are calculated according to column.

NA, not applicable; T2DM, type 2 diabetes mellitus; SAT, Systemic arterial hypertension; BMI, body mass index; CFU, colony-forming unit; WS, waist size; SC, serum creatinine; UAlb, urinary albumin; GFR, glomerular filtration rate; Glu, fasting serum glucose.

* Determined in 41 subjects of no renal impairment group and 30 subjects of with renal impairment group.

† Units: cm. Determined in 25 females of no renal impairment group and 23 females of with renal impairment group and in 14 males of NRI and six males of WRI group.

‡ Units: mg/dL.

§ Units: mL/min/1.73 m^2^.

╗ CLED agar per milliliter.

The groups with renal impairment and no renal impairment were compared in terms of their anthropometric, biochemical characteristics and CFU in CLED agar. No differences were found between the groups in terms of weight, height, BMI, waist size in general, female and male subgroups, CFU and serum fasting glucose. In contrast, creatinine in general, female and male subgroups and albumin were higher in the group with renal impairment, while GFR was greater in the group no renal impairment (Table 1). By definition, only the subjects of the group with renal impairment presented microalbuminuria and none of the subjects included presented macroalbuminuria. A total of 19 subjects presented microalbuminuria: one subject corresponded to stage 1; eight subjects to stage 2 and 10 subjects to stage 3.

In addition, the groups were compared in terms of the presence of CO, OW and T2DM duration (<10 years vs. ≥10 years). No differences were found between groups in any of these parameters. UTI was identified in 17 subjects, all females; 9/42 (21%) in the group no renal impairment and 8/34 (24%) in the group of with renal impairment. There were no differences between groups (*P* = 0.83).

A comparison was made among the frequencies in the results of the variables of the reagent strips for urinalysis. All subjects presented a normal result to urobilinogen and specific gravity. No differences were found in leukocytes, nitrite, protein, ketone, glucose and pH between the no renal impairment and with renal impairment groups. In contrast, differences were found in bilirubin, in the no renal impairment group, where none of the subjects presented a positive result for bilirubin compared to the with renal impairment group with 4/34 (12%) (*P* = 0.04).

### Urinary MMP-9 measurements

ELISA was used to determine urinary MMP-9 levels. Based on these values, samples were categorized according to P_90_. There was a correlation between urinary MMP-9 levels and period of time in years with T2DM diagnosis (Rho = 0.315, *P* = 0.006), CFU (Rho = 0.365, *P* = 0.001), and with stage of CDK (Rho = 0.243, *P* = 0.03). No correlations were found between urinary MMP-9 levels and age, BMI, waist size, number of medicines taken to treat T2DM and fasting serum glucose. Correlation of urinary MMP-9 with GFR was found with a borderline *P*-value (Rho = −0.205, *P* = 0.07).

No differences were found (*P* = 0.06) in the levels of urinary MMP-9 between the no renal impairment (median 0.0, P_25_ 0.0, P_75_ 0.6) and with renal impairment (median 0.09, P_25_ 0, P_75_ 1.18) groups. The absence or presence of MMP-9 and values of P_90_ were compared among subjects in the groups no renal impairment and with renal impairment and a sub-analysis conducted excluding subjects with UTI. The presence of MMP-9 (*P* = 0.02) and that of its P_90_ (*P* = 0.04) were associated with the with renal impairment group; however, in the sub-analysis results, the presence of MMP-9 (*P* = 0.05) was associated with the with renal impairment group only, but not with its P_90_ (*P* = 0.58) (Table 2).

**Table 2:** Levels of urinary MMP-9 of participants, general and without urinary tract infection, according to renal impairment status.

MMP status	General *n* = 76		No renal impairment *n* = 42		With renal impairment *n* = 34		*P*-value
	*n*	%	*n*	%	*n*	%	
MMP-9 absent	31	(41)	22	(52)	9	(26)	0.02
MMP-9 present	45	(59)	20	(48)	25	(74)	
MMP-9 <P_90_^†^	69	(91)	41	(98)	28	(82)	0.04
MMP-9 ≥P_90_^†^	7	(9)	1	(2)	6	(18)	

	Without UTI *n* = 59		No renal impairment *n* = 33		With renal impairment *n* = 26		
MMP-9 absent	29	(49)	20	(61)	9	(35)	0.05
MMP-9 present	30	(51)	13	(39)	17	(65)	
MMP-9 <P_90_^†^	56	(95)	32	(97)	24	(92)	0.58
MMP-9 ≥P_90_^†^	3	(5)	1	(3)	2	(8)	

**Notes:**

Values are given as frequencies (percentage). All percentages are calculated according to column.

UTI, urinary tract infection; P, percentile.

† Percentile 90 = 6.0684 ng/ml.

The presence of MMP-9 (*P* = 0.006) was associated with UTI 15/17 (88%), compared to those without UTI 30/59 (51%). There was also a greater level of MMP-9 (*P* = 0.001) in subjects with UTI (median 0.597, P_25_ 0.0713, P_75_ 5.652), compared to those without (median 0.0110, P_25_ 0, P_75_ 0.304). Levels of MMP-9 (*P* < 0.001) were greater in the women (median 0.231, P_25_ 0.0118, P_75_ 2.247) compared to the men (median 0, P_25_ 0, P_75_ 0).

### Multivariate analysis of factors associated with microalbuminuria

Four multivariate models were constructed in order to identify the association between MMP-9 (independent variable) and renal impairment (dependent variable), introducing up to presence of UTI, presence of nitrites in urine, duration of T2DM (≥15 years), age > 60 years and sex (male). In unadjusted models 1 and 2, the variables showed no statistically significant association. In contrast, in both of the adjusted models, the presence of MMP-9 (*P* = 0.02) and its P_90_ (*P* = 0.05) showed an association with renal impairment. The remaining variables were not found to be significantly associated (Table 3).

**Table 3:** Multivariate analysis of the association between MMP-9 with renal impairment.

Covariates	Unadjusted model			Adjusted model		
	OR	CI 95%	*P*-value	OR	CI 95%	*P*-value
**Model 1**						
MMP-9 present	2.2	0.7–7.6	0.20	3.1	1.2–8.1	0.02
UTI	0.4	0.1–1.8	0.25	–	–	0.59
Positive nitrites	9.1	0.8–101.9	0.07	–	–	0.09
T2DM ≥ 15 years	1.7	0.6–4.7	0.34	–	–	0.26
Age > 60 years	2.0	0.7–5.6	0.20	–	–	0.08
Sex (male)	0.7	0.2–2.4	0.52	–	–	0.86
**Model 2**						
MMP-9 ≥P_90_	3.1	0.3–36.7	0.37	8.9	1.002–77.0	0.05
UTI	0.5	0.1–2.1	0.34	–	–	0.67
Positive nitrites	5.5	0.4–79.7	0.21	–	–	0.36
T2DM ≥ 15 years	1.7	0.6–4.8	0.32	–	–	0.22
Age > 60 years	2.1	0.8–5.9	0.15	–	–	0.10
Sex (male)	0.5	0.1–1.4	0.18	–	–	0.26

**Notes:**

Odds ratios were obtained by logistic regression. Covariates were introduced as categorical variables. Unadjusted model: variables were introduced using the enter method. Adjusted model: variables were introduced using the forward conditional method. Odds ratios were not calculated in the adjustment variables.

P_90_, Percentile 90; UTI, urinary tract infection; T2DM, type 2 diabetes mellitus.

## Discussion

This study revealed in multivariate analysis that the presence of urinary MMP-9 and its P_90_ is associated with renal impairment in Mexican patients with T2DM. To our knowledge, this is the first study to utilize ELISA levels of MMP-9 in urinary samples taken from T2DM patients with access to social security in Mexico. While there are several reports of MMP-9 in kidney diseases (Chang et al., 2006; Rysz et al., 2007; Peiskerova et al., 2009; Van Der Zijl et al., 2010; Bojic et al., 2015; Gluba-Brzozka et al., 2016; Hernandez-Hernandez et al., 2017), as a function of the subjects included, the methodology employed and the fluid analyzed (urine), there are few with which we can compare our findings. To our knowledge, the present study could be compared directly to those of Altemtam, Nahas & Johnson (2012), Thrailkill et al. (2010) and Han et al. (2008). Further studies are required in order to determine the origin of the MMP-9, which is probably from tubule or leukocyte cells, urinary enumeration analysis also should be conducted.

The study by Altemtam, Nahas & Johnson (2012), while differing in terms of the inclusion of diabetic kidney disease subjects with macroalbuminuria and at stages 3–4, according to the K/DOQI guide, demonstrated that urinary gelatinase activity attributable to MMP-2 and MMP-9 in DM patients with diabetic kidney disease is 25% greater, compared to that of those with DM alone. In our study, while in the continuous value of urinary MMP-9 there were no differences found between T2DM patients with and without renal impairment, the presence, and the P_90_, of this gelatinase was associated with the with renal impairment group, but not with the no renal impairment group. Altemtam, Nahas & Johnson (2012) found that urinary gelatinase activity attributable to MMP-2 and MMP-9 in DM patients is threefold higher than in apparently healthy volunteers, and that total urinary activity of MMP-1, MMP-2, MMP-8, MMP-9, MMP-13/creatinine ratio is significantly higher in females compared to males, irrespective of albuminuria. In our study, higher levels of urinary MMP-9 were associated with females, compared to males.

Thrailkill et al. (2010) demonstrated in T1DM subjects (without other chronic inflammatory disease, autoimmune disease, malignancy or UTI) that urine MMP-9 is significantly higher in females compared to males. This group also reported that urine MMP-9/creatinine concentrations displayed a menstrual cycle-specific pattern, and that this pattern is not simply a consequence of menstrual cycle fluctuations in glomerular protein permeability, thus there are no differences in urinary albumin excretion among menstrual cycles (Thrailkill et al., 2010). Thrailkill et al. (2010) propose that sex-specific differences could support a contribution to susceptibility for development of renal complications in T1DM. This is interesting since the pattern of urinary MMP-9 in relation to sex reported by Thrailkill et al. (2010) in T1DM subjects, appears to coincide with that of Altemtam, Nahas & Johnson (2012) in DKD subjects with macroalbuminuria at stages 3–4, and with the findings of the present study in subjects with T2DM, with and without renal impairment.

In a cross sectional study of adults, Han et al. (2008) found that urinary MMP-9 is associated with acute kidney injury (AKI), but cannot not differentiate subjects with AKI from those with UTI. Han et al. (2008) also reported that levels of urinary MMP-9 are significantly higher in UTI compared to AKI and healthy controls. In the study of Han et al. (2008) UTI was defined as present in patients without evidence of previous renal disease or AKI, positive leukocyturia and urine culture greater than 10^5^ UFC. While our definition of UTI differs from that of Han et al. (2008), and our study was focused on subjects with T2DM with and without renal impairment, in whom GFR was measured on a single occasion, we found differences in the levels of MMP-9 between subjects with and without UTI. However, in the multivariate analysis, the presence of UTI was not associated with the presence of renal impairment.

Chronic kidney disease is associated with alterations in primary host defense mechanisms and increases the risk of bacterial infections. Epidemiological evidence shows that UTI, pneumonia and sepsis are the most common infectious complications in CKD (Kidney Disease: Improving Global Outcomes (KDIGO) CKD Work Group, 2013). Due to the fact that kidney disease is a public health problem, accurate screening for proteinuria in high-risk groups has been proposed (Keane & Eknoyan, 1999; Kidney Disease: Improving Global Outcomes (KDIGO) CKD Work Group, 2013). In this sense, determination of proteinuria/albuminuria in subjects with UTI is controversial; on one hand, there are reports that is can change (National Kidney Foundation, 2002; Kwak, Chung & Kim, 2010), and on the other, Carter et al. (2006) reports no evidence of association between asymptomatic UTI and proteinuria. Moreover, there is evidence in DM patients that asymptomatic bacteriuria does not cause albuminuria (Carter et al., 2006). Further studies are required to fully validate the predictability of proteinuria/albuminuria in UTI patients.

This study presents various limitations; due to the low sensivity of the ELISA kit, the absence of MMP-9 found in urinary samples could be questionable. A further limitation is the absence of biopsy and lack of measurement of urinary creatinine, HbA1c, HDL, LDO and BUN. However, the strength of this study is the type of analysis employed, which allowed adjustment for potential confounding factors.

One of the probable mechanisms by which MMP-9 could contribute to kidney fibrosis in CDK has been explored in BALB/c genetically modified mice, showing that in primary mouse renal peritubular endothelial cells, the endothelial–mesenchymal transition via notch signaling is MMP-9 dependent (Zhao et al., 2016). In human kidney glomerular endothelial cells in culture, MMP-9 induces endothelial-mesenchymal transition via notch activation (Zhao et al., 2016). Moreover, in Zucker diabetic rats there is increased glomerular MMP-9 staining in hyperplastic parietal epithelial cells that is often accompanied by decreased staining for podocyte markers in the sclerotic area of affected glomeruli (Zhang et al., 2015).

## Conclusions

Sensitive and non-invasive biological markers of kidney injury are required for the early detection of KD. In this study, we demonstrate that the presence and P_90_ of urinary MMP-9 are associated with renal impairment in Mexican patients with T2DM.

## Supplemental Information

10.7717/peerj.6067/supp-1 Supplemental Information 1 Raw data.

## Additional Information and Declarations

### Data Availability

The following information was supplied regarding data availability: The raw data are provided in a Supplemental File.
